# Understanding fracture populations by epidemiology

**DOI:** 10.1080/17453674.2020.1763571

**Published:** 2020-05-12

**Authors:** Antti P Launonen

**Affiliations:** Department of Orthopaedics and Traumatology, Tampere University Hospital, Teiskontie, Tampere, Finland

In orthopedics, we tend to classify and quantify different traumas in order to focus our efforts and resources on the specific areas of need. To gain a perspective, we use epidemiology as a fallback instead of trusting on our own feelings of prevalence. There are several ways to access the analysis performed in epidemiological studies. For example, hospital discharge registries are usually nationwide or form part of a subsample of a larger area patient cohort; they give a reliable overview of the data attained. There are, however, several problems with such registries. One major problem is the inability to access patient-level information. This leads to only the overall numbers of incidences and rates of interventions being provided, and a subsequent lack of incident classification and patient reported outcome measures (PROMs). Another way to attain the data is a cohort sample of the catchment population of a specific area. The problem with this method is the relative rarity of the specific cases (i.e., fractures). In addition, it is time consuming to accumulate a large enough cohort for analysis. Furthermore, the data provided are usually retrospective in nature and can lead to deficiencies and heterogeneity of the dataset.

The study by Court-Brown et al. (2001) reported the 5-year data of all consecutive proximal humeral fracture patients, including both out-patients and in-patients, treated at the Trauma Unit of a hospital in Edinburgh, Scotland (Court-Brown et al. 2001). At the time, the catchment area for the fracture population was 700,000, which could be estimated to be sufficient for a meaningful cohort for relevant analysis of the fractures. The strength of the study that makes it unique is its prospective nature. As a result, the data acquired were comprehensive with detailed patient demographics and radiographic images for fracture classification. The incidence data implied full coverage and stratified by age and were included adjacent, patient-matched fracture classification.

The study raised several important issues. For example, the fractures that occurred in young patients were mainly related to sports or high velocity injuries, whereas those that occurred in patients 30 years and older were mostly caused by falling from a standing height. The authors could show the peak incidence for older patients, which was also associated with more severe fracture patterns than in younger patients. Furthermore, they were able to classify all the patients using the Neer and AO classification systems (Neer 1970, Müller et al. 1990). Of the 2 systems used, the AO-classification was shown to be more comprehensive. The authors acknowledged, however, that there were problems in intra- and interobserver reliability, which was later rated as moderate for both the Neer and AO classifications (Papakonstantinou et al. 2016). However, they raised this important issue for discussion:

“Neer three- and four-part fractures and fracture dislocations and the AO type C fractures occurred with an incidence of 13% and 6%, respectively, indicating that the literature does not adequately respect the spectrum of proximal humeral fractures”

In our smaller, retrospective cohort study from year 2015, we found a similar rate of fractures classified with Neer for three- and four-part fractures with an incidence of 19% and 7%, respectively (Launonen et al. 2015). Therefore, the veracity of the statement still stands. Naturally, such multipart fractures cause the most difficulty for clinicians when trying to make treatment decisions.

The Court-Brown study has aged well, and it can still be considered as a benchmark of proximal humeral fracture epidemiology with large prospective patient cohorts. In the clinical and research field, we need detailed epidemiological data so that we can focus on the key issues. However, the data provided in the study need an update since the age distribution has changed over time, which may affect the fracture distribution. Although the epidemiology and fracture classification do not help us decide on the best treatments for patients, they do help us to understand the disparity between the common and the rare and act as a baseline in the guidelines (Brorson et al. 2012). Thus, they help us to better focus our efforts and resources on the specific areas of need.

Antti P Launonen *Department of Orthopaedics and Traumatology,* *Tampere University Hospital, Teiskontie, Tampere, Finland* *E-mail:*antti.launonen@pshp.fi
